# Recent Insights into the Biomarkers, Molecular Targets and Mechanisms of Non-Alcoholic Steatohepatitis-Driven Hepatocarcinogenesis

**DOI:** 10.3390/cancers15184566

**Published:** 2023-09-14

**Authors:** Anna Kakehashi, Shugo Suzuki, Hideki Wanibuchi

**Affiliations:** Department of Molecular Pathology, Osaka Metropolitan University Graduate School of Medicine, 1-4-3 Asahi-machi, Abeno-ku, Osaka 545-8585, Japan; shugo@omu.ac.jp (S.S.); wani@omu.ac.jp (H.W.)

**Keywords:** NAFLD, NASH, HCC, biomarker, hepatocarcinogenesis mechanisms

## Abstract

**Simple Summary:**

This review is focused on the research recently published in the field of non-alcoholic fatty liver disease (NAFLD) or metabolic dysfunction-associated steatotic liver disease (MASLD), and steatohepatitis (NASH)-associated liver cancer. We aimed to highlight recent omics research on novel genetic and protein contributions to NAFLD/NASH to discuss the mainstream molecular pathways and novel candidate biomarkers and molecular targets in NAFLD/NASH. Special attention is paid to the role mTOR, as multifunctional orchestrator in NASH progression to HCC.

**Abstract:**

Non-alcoholic fatty liver disease (NAFLD) or metabolic dysfunction-associated steatotic liver disease (MASLD) and steatohepatitis (NASH) are chronic hepatic conditions leading to hepatocellular carcinoma (HCC) development. According to the recent “multiple-parallel-hits hypothesis”, NASH could be caused by abnormal metabolism, accumulation of lipids, mitochondrial dysfunction, and oxidative and endoplasmic reticulum stresses and is found in obese and non-obese patients. Recent translational research studies have discovered new proteins and signaling pathways that are involved not only in the development of NAFLD but also in its progression to NASH, cirrhosis, and HCC. Nevertheless, the mechanisms of HCC developing from precancerous lesions have not yet been fully elucidated. Now, it is of particular importance to start research focusing on the discovery of novel molecular pathways that mediate alterations in glucose and lipid metabolism, which leads to the development of liver steatosis. The role of mTOR signaling in NASH progression to HCC has recently attracted attention. The goals of this review are (1) to highlight recent research on novel genetic and protein contributions to NAFLD/NASH; (2) to investigate how recent scientific findings might outline the process that causes NASH-associated HCC; and (3) to explore the reliable biomarkers/targets of NAFLD/NASH-associated hepatocarcinogenesis.

## 1. Introduction

**Figure 1:**
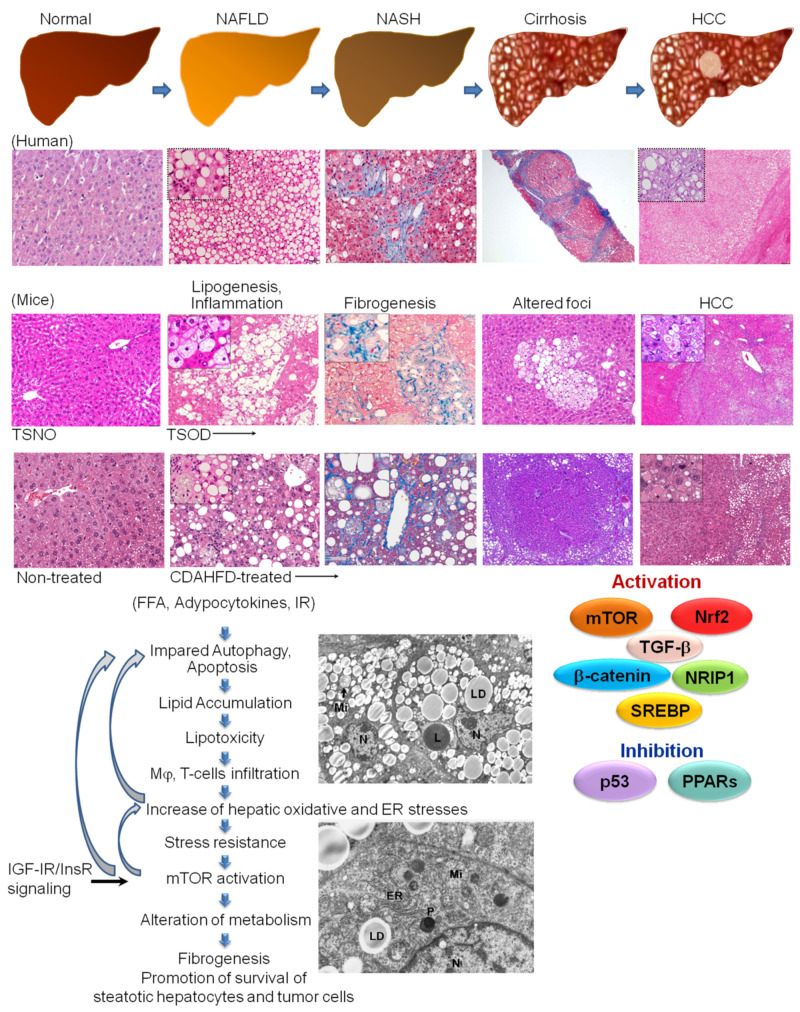
The developmental process of NAFLD/NASH-associated HCC in human and NASH model mice. The collagen-rich fibrotic regions are visualized via Masson’s trichrome staining. Transmission electron microscopy (TEM) is performed in TSOD mice HCC. LD: lipid droplet, Mi: mitochondria, N: nuclei, ER: endoplasmic reticulum, P: peroxisome, L: lysosome.

The global epidemic of obesity and metabolic syndrome has become a significant threat to human health worldwide. Recent epidemiological evidence shows that non-alcoholic fatty liver disease (NAFLD), recently termed metabolic dysfunction-associated steatotic liver disease (MASLD), and steatohepatitis (NASH) have rapidly become leading etiologies predisposing to fibrosis, cirrhosis, and hepatocellular carcinoma (HCC) development due to the rapid increase in metabolic syndrome and obesity in the past decades and newly available therapies for hepatitis C (HCV) and B virus (HBV)-associated HCC [1,2,3,4] (Figure 1). NAFLD/NASH-HCC is known to have even lower survival rates than viral hepatitis HCC and is generally developed based on metabolic syndrome, hyperinsulinemia, insulin resistance, and dyslipidemia, and is characterized by fat accumulation in the liver, either due to the increased inflow of free fatty acids or de novo lipogenesis [3,4]. NAFLD, a hepatic manifestation of insulin resistance, is very important in the pathophysiology of type 2 diabetes (T2DM). Furthermore, T2DM confers a three-fold risk of HCC [5]. However, in recent studies, it was reported to occur in cirrhosis-free and non-obese patients (“lean NAFLD”) and to be triggered by endotoxin-related inflammation in the gut and adipocytokines, leptin, and adiponectin [4,6,7,8].

As for the mechanism, recently, the “multiple-parallel-hits” hypothesis was proposed, in which NASH was suggested to occur upon chronic liver damage caused by abnormal metabolism and lipotoxicity, lipid peroxidation, mitochondrial dysfunction, oxidative, endoplasmic reticulum stresses, inflammation, and cell death [9,10].

Monitoring the degree of hepatic necrotizing inflammation and fibrosis is crucial to predicting the long-term prognosis of NAFLD patients. Early intervention is necessary to slow disease progression. While liver biopsy is the gold standard for diagnosis, it is not widely used due to its invasive nature. Additionally, alpha-fetoprotein (AFP) is commonly used to diagnose HCC in NASH patients, but recent studies have found it difficult to distinguish between NASH and NASH-HCC patients [11,12]. Therefore, novel biomarkers are urgently needed for accurate and early NASH-HCC diagnosis and prognosis. The emergence of genomics, transcriptomics, proteomics, metabolomics, and lipidomics provides an opportunity to gain insight into multiple processes that contribute to the development of non-alcoholic steatohepatitis (NASH). Moreover, these technologies can aid in the identification of novel biomarkers for the diagnosis and prognosis of non-alcoholic fatty liver disease (NAFLD), NASH, and hepatocellular carcinoma (HCC). On the other hand, the discovery of NAFLD/NASH-associated physiopathological signaling cascades has benefited from large-scale omics studies describing the modulation of genes, proteins, and metabolites. In addition to well-known regulators of NAFLD/NASH hepatocarcinogenesis, such as transforming growth factor beta (TGF-β), SMAD family member 3 (SMAD3), insulin-like growth factor 1 (IGF1), peroxisome proliferation activating receptors (PPARs), sterol regulatory element-binding protein and liver X receptor α (SREBP-LXRα), CCAAT/enhancer-binding protein beta (CEBPB), nuclear factor κ-light-chain-binding protein (NFkB), c-myc, Wnt/β-catenin, signal transducer and activator of transcription 3 (STAT3), and p53, recent research has highlighted the important role of protein kinases mammalian target of rapamycin (mTOR), protein receptor sequestosome 1 (SQSTM1, p62), PI3K/Akt, PKC, MAPK, ErbB, nuclear factor (erythroid-derived 2)-like 2 (Nrf2), hepatocyte nuclear factor 1α (HNF1α) and 4α (HNF4α), and nuclear-receptor-interacting protein 1 (NRIP1; nuclear factor RIP140) in this process [12,13,14,15,16,17].

Our primary focus was on the omics studies’ contributions to human and NASH animal models. These studies offer the potential for identifying novel, relevant biomarkers that can aid in predicting and prognosing NASH hepatocarcinogenesis in NAFLD-associated HCC while also enhancing our understanding of the molecular basis of the disease.

## 2. Signaling and Metabolic Pathways in the Regulation of NAFLD/NASH Progression, Hepatocarcinogenesis, and Associated Biomarkers

### 2.1. mTOR Pathway as a Multifunctional Driver of Metabolic Syndrome-Associated Hepatocarcinogenesis

**Figure 2:**
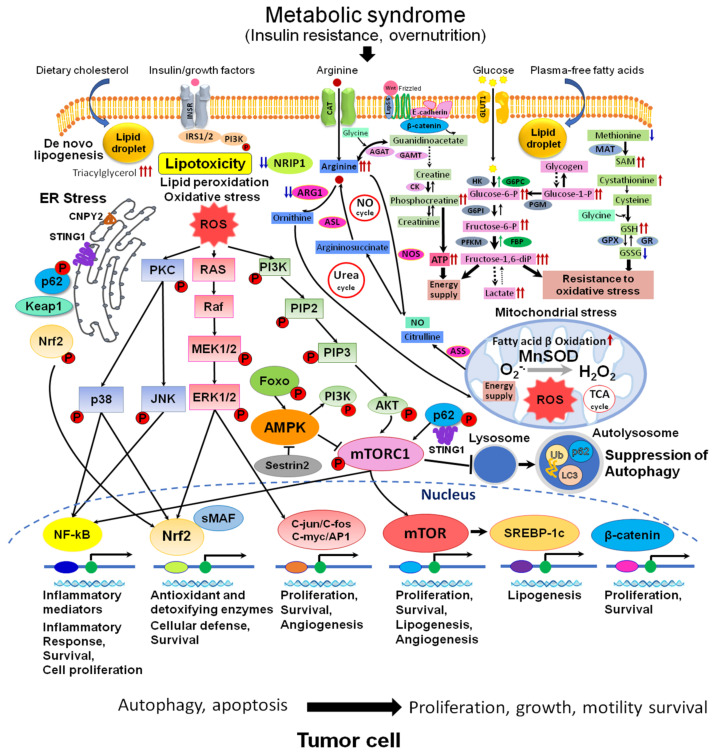
Metabolic and signaling pathways in NAFLD/NASH-associated HCC. Abnormal metabolism: accumulation of arginine, glucose metabolites, phosphocreatine, SAM, GSH, and alterations of urea and NO cycles are observed in NAFLD/NASH HCC. These events are associated with developed lipotoxicity, activation of de novo lipogenesis (SREBP-1), lipid peroxidation, fatty acid β oxidation, mitochondrial dysfunction, activation of oxidative (MAPK, Nrf2, NF-kB, C-jun/C-fos/C-myc/AP1) and ER stresses (p62, Keap1, Nrf2, STING, CNPY2)-related signaling, wnt/β-catenin pathway, and suppression of autophagy (mTORC1, AMPK, p62), with the central role for mTOR pathway. Abbreviations: ROS, reactive oxygen species; NO, nitric oxide; ER, endoplasmic reticulum; TCA, tricarboxylic acid cycle; INSR, insulin receptor; GLUT1, glucose trasporter 1; STING1, stimulator of interferon response cGAMP interactor 1; SAM, S-adeninemethyonine; SAH, S-adenosyl-L-homocysteine; Ub, ubiquitin; LC3, microtubule-associated protein 1A/1B−light chain 3; sMAF, small maf protein; CAT, cationic amino acid transporter; PKC, protein kinase C; MnSOD, manganese superoxide dismutase; PIP2, phosphatidylinositol 4,5-bisphosphate; PIP3, phosphatidylinositol 3,4,5-triphosphate; PI3K, phosphoinositide 3-kinase; AKT, protein kinase B (serine/threonine-specific protein kinase); p38, p38 mitogen-activated protein kinase; MEK, mitogen-activated protein kinase kinase; ERK, extracellular signal-regulated kinase; JNK, c-Jun N-terminal kinase; ARG1, arginase1; ASL, argininosuccinate lyase; ASS, argininosuccinate synthase; AGAT, arginine-glycine amidinotransferase; GAMT, guanidinoacetate N-methyltransferase; NOS, nitric oxide synthase; CK, creatine kinase; HK, hexokinase; G6PI, glucose-6-phosphate isomerase; PFKM, 6-phosphofructokinase; G6PC, glucose-6-phosphatase; FBP, fructose 1,6-bisphosphate aldolase; PGM, phosphoglycerate mutase; MAT, methionine adenosyltransferase; GPX, glutathione peroxidase, GR, glutathione reductase.

Numerous clinical studies have established a causal connection between metabolic syndrome and diabetes with various types of cancer, including HCC. These conditions heighten the risk of developing cancer and facilitate the process of hepatocarcinogenesis by activating inflammatory pathways that generate proinflammatory cytokines and reactive oxygen species (ROS). This, in turn, leads to genomic instability, cellular proliferation, and the suppression of apoptosis [18] (Figure 2). The accumulation of lipids in the liver is a significant contributor to the progression of NAFLD in individuals with T2DM. The mTOR signaling pathway has a crucial role in regulating metabolic processes in various organs, including hepatic lipid metabolism, insulin resistance, chronic liver inflammation, and, ultimately, the advancement of NAFLD [18].

The protein kinase mTOR is the prime focus of rapamycin, an immunosuppressive drug that operates via two distinct complexes. These are mTORC1, which incorporates the regulatory-associated protein of mTOR (Raptor) and the rapamycin-associated TOR protein, and mTORC2, which consists of the rapamycin-insensitive companion of mTOR (Rictor). As there are two key regulatory components, the regulation of mTOR is very complex and is carried out by various proteins, hormones, growth factors, and nutrients [19]. mTOR controls, at least in part, four major metabolic processes responsible for the regulation and maintenance of normal levels of hepatic triglycerides (TG) amounts, which are altered in individuals with insulin resistance, obesity, and diabetes mellitus [20]. These include the delivery of plasma albumin-bound fatty acids (FAs), transferred from adipose tissue, TG synthesis from acetyl-coenzyme A (Ac-CoA) derived mainly from the metabolism of glucose in mitochondria, FAs oxidation, and TG secretion in very-low-density lipoprotein (VLDL) [19].

In recent years, protein kinases, including mTOR, MAPK, PKC, PI3K/Akt, and ErbB, were shown to govern most of the pathological pathways in NAFLD and regulate both inflammation and hepatic gluco- and liponeogenesis [21] (Figure 2). In connection with lipid metabolism alterations in NASH HCCs, proteomics research revealed overexpression of the downstream proteins of the SREBP-LXRα pathway (e.g., cytochrome P450 isoenzymes CYP51A1, CYP4F11 and CYP8B1, cortisone, etc.), and inhibition for those involved in lipid catabolism, such as molecules downstream of peroxisome proliferator-activated receptors α (PPARα) and γ (PPARγ), as well as mitochondrial and peroxisomal proteins involved in the process of oxidation of fatty acids (e.g., enoyl-CoA hydratase (ECHS1), 3-ketoacyl-CoA thiolase (ACAA2), and fatty acid binding protein 2 (FABP1)) [12,13]. In addition, the study found that NASH-HCC had increased lipid, cholesterol, and bile acid biosynthesis, elevated levels of triacylglycerol, and higher levels of AFP, AST, and cancer antigen 19-9 compared to NASH liver [12]. The pivotal role of mTOR in the regulation of metabolism, lipid biosynthesis, fibrogenesis, and NAFLD/NASH progression was confirmed with the help of omics techniques, and the close relationship with IGF1, TGF-β/SMAD, SREBP, PPARs, and other important cellular regulators was reported in human and animal studies [13,14,15]. Recent reports highlighted the crosstalk between mTOR and its upstream regulators such as Notch, Hedgehog, and Hippo [22]. It also delves into the mechanisms of the mTOR regulation of various factors, like Foxo1, Lipin1 (insulin resistance), oxidative stress (PIG3, p53, JNK), SREBPs (lipogenesis), Toll-like receptors (TLRs) (gut microbiota), autophagy, inflammation, genetic polymorphisms, and epigenetics in NAFLD [22]. Serum metabolic profiling further revealed the alteration of metabolites associated with NASH HCC and implicated in the regulation of the metabolism of lipids by PPARα, fatty acid metabolism, biogenic amine synthesis, downregulation of necroptosis, amino acid metabolism, and mTOR signaling pathway [12]. Proteome and metabolome analyses revealed the activation of mTOR in conjunction with AKT, PI3K, extracellular signal-regulated kinase 1/2 (ERK1/2) kinases, and β-catenin in the liver tumors developed in Tsumura, Suzuki, Obese Diabetic (TSOD) metabolic syndrome/T2DM NASH model mice [14,15] (Figure 2). This was also confirmed in human NASH HCCs [14]. However, mTOR activation was not prominent in CDAHFD- and DEN-treated mice and human virus-associated HCC [15]. When the proteomes of human NASH and HCV-associated HCCs were compared, altered proteins and enzymes involved in lipid, cholesterol, and bile acid biosynthesis, fatty acid oxidation and catabolism, mitochondrial dysfunction, and the downstream molecules of activated SREBP-LXRα, NRIP1, and inhibited PPARs was specific to NASH, but not the virus-associated HCC [14]. From these data, we conclude that along with IR and activation of TGF-β, the dysregulation of Wnt/β-catenin, NRIP1, Nrf2, SREBP-LXRα, PHBs, PPARs, and p53 play an important role in NASH pathogenesis. These findings caused an impulse in the discovery of novel biomarkers of NASH HCC and numerous chemopreventive and therapeutic studies with mTOR inhibitors in NAFLD/NASH/HCC [23].

Through studies investigating mTOR biomarkers and inhibitors, it has been revealed that mTOR plays a critical role in regulating adipocyte and lipogenic marker genes, including PPAR and SREBP1c. These genes are essential for lipogenesis and reducing inflammation and fibrosis. mTOR activation also influenced lipid biosynthesis in the liver preneoplastic (altered) foci and tumors of NASH model mice, inducing the tremendous accumulation of lipid droplets in the cells (ballooning) [14,15]. The discovery of hepatic G protein-coupled receptor 180 (Gpr180) has revealed its significant impact on lipid metabolism. It has been observed to increase triglyceride and cholesterol levels in the liver and plasma, leading to hepatic lipid deposition in HFD-treated Gpr180 knockout (KO) mice. Additionally, Gpr180 has been shown to decrease energy metabolism and activate adiposity [24] (Table 1 and Table 2). Knockdown of Gpr180 in the Huh7 hepatoma cell line resulted in the downregulation of mTORC1 signaling, as detected by transcriptome analysis. Furthermore, decreases in activated SREBP1 cholesterol homeostasis and amelioration of plasma and hepatic lipid levels were observed in Gpr180 knockout mice. The study confirmed the relationship between Gpr180 and mTOR, SREBP1, and SREBP2. As a result, it was determined that Gpr180 could serve as a promising NAFLD biomarker and drug target for the treatment of NAFLD/NASH [24]. Another potential NAFLD/NASH biomarker and potential molecular target is the stimulator of interferon response cGAMP interactor 1 (STING1) [25] (Table 1 and Table 2). Recent research indicates that STING1 plays a crucial role in the activation of MTORC1 by palmitic acid. The interaction of STING1 with various components of the MTORC1 complex and its reliance on SQSTM1 (p62), a crucial regulator of the MTORC1 pathway, enable this accomplishment. The activation of MTORC1 and STING1 were observed together in the liver tissues of NAFLD patients, providing clinical evidence that STING1 is involved in the activation of MTORC1 [25].

Concerning NASH hepatocarcinogenesis, it is also important to mention the recently discovered novel potential prognostic biomarker of human NAFLD/NASH-HCC, sphingosine 1-phosphate receptor 2 (S1PR2) (Table 1 and Table 2). The protein serves as a receptor on the cell membrane for sphingosine 1-phosphate (S1P), a lysophospholipid that is biologically active and has diverse physiological effects [26]. S1PR2 is responsible for the progression from NAFLD to NASH. The association of conjugated bile acids (CBAs) and S1PR2 alterations with the onset of NASH hepatocarcinogenesis was found in human NAFLD/NASH-HCCs in vivo and in Huh 7 and HepG2 hepatoma cell lines [26]. The cell cycle phases G1/G2 were regulated by S1PR2, which had a significant impact on the proliferation, invasion, migration, and apoptosis of both Huh 7 and HepG2 cells. Bioinformatics analysis further revealed a strong correlation between S1PR2 and several tumor-related pathways, including PI3K/AKT/mTOR, JAK/STAT, NF-kB, and ERK. Notably, high expression of S1PR2 was associated with poor prognosis in patients with NAFLD/NASH-HCC [26].

Another potential prognostic marker in metabolic syndrome/NASH-associated human liver cancer is arginase 1 (ARG1), whose expression is downregulated in NAFLD/NASH-HCC (Table 1 and Table 2). ARG1 suppression was bound to shift from arginine to the production of guanidinoacetate and phosphocreatine (Figure 2). The study found that there is a strong correlation between low ARG1 expression and poorly differentiated tumors, higher pathological stage, and decreased patient survival, particularly in those with metabolic syndrome/T2DM. Suppression of ARG1 was associated with mTOR activation [14]. High expression of ARG1 was observed in HCV-positive HCC, while it was low in tumors associated with metabolic syndrome/NASH. Moreover, poor differentiation of NASH HCC was found to be primarily correlated with ARG1 negativity, which could explain the lower survival rates in patients.

### 2.2. Disturbances of Metabolism in NAFLD/NASH-HCC

Untargeted metabolomics has been recently applied to find novel and peculiar metabolite profiles in NADLD/NASH/HCC patients and NASH model mice and to give insights into discovering new biomarkers in NASH pathophysiology. The liver of TSOD NASH model mice showed an increase in lipogenesis, inflammation, peroxisome proliferation, and fibrogenesis, along with a significant rise in glucose metabolites, including glucose 1-phosphate, glucose 6-phosphate, galactose 1-phosphate, fructose 6-phosphate, and primarily fructose 1,6-diphosphate, in HCCs [14] (Figure 2). It was found that TSOD mice with liver preneoplastic lesions and tumors had significantly increased levels of polysaccharides, along with a drastic increase in amino acid L-arginine and notable increases in phosphocreatine, S-adenosylmethionine/S-adenosylhomocysteine ratio, and adenylate and guanylate energy charges [14].

Masarone et al. conducted a recent human metabolomics study that analyzed 307 subjects with steatosis, NASH, and NASH-cirrhosis. Through the study, they were able to identify specific metabolites that distinguished between the different patient classes [52]. In this investigation, an elevation of glycocholic acid, taurocholic acid, phenylalanine, and branched-chain amino-acids accompanied by a decrease in glutathione was found with the increase in the severity of the disease from steatosis to NASH and NASH-cirrhosis. Very recently, Ahmed et al. reported a depletion of amino acid leucine in NASH-HCC as compared with NASH patients [12] (Table 1 and Table 2). In the past, branched-chain amino acids have been effective in inhibiting the growth and angiogenesis of HCC through the suppression of insulin resistance and degradation of vascular endothelial growth factor mRNA [12,53]. Significantly, they were found to trigger apoptosis in liver cancer cells by disrupting mTOR-related pathways [54]. Metabolomics profiling has proven to be an effective non-invasive diagnostic tool for distinguishing between NAFLD/NASH-HCC and identifying the different stages of the disease [14,52].

The emerging area of NAFLD/NASH-associated cancer development and progression was devoted to research on lipid metabolism [55]. The risk of NAFLD was reported to be increased with an imbalance between fatty acid uptake, oxidation, and secretion and a high level of plasma phospholipids (e.g., phosphatidylethanolamine), which can act as signal transductors with oncogenic and tumor-suppressive roles [12]. In addition, suppression of mitochondrial β-oxidation with increased peroxisomal and microsomal ω-oxidation accompanied by accumulation of hepatic fatty acids in NASH resulted in the activation of lipotoxicity and progression of the disease.

The HCCs in TSOD mice showed alterations in intracellular pathways, such as suppression of the urea cycle, methionine and putrescine degradation pathways, and an increase in cellular antioxidants like glutathione (GSH) levels. This indicates a boost in antioxidant activity in the HCCs [13]. Proteome analysis of HCCs revealed that increased levels of L-arginine significantly inhibited urea cycle enzymes, such as ARG1, argininosuccinate lyase (ASL), argininosuccinate synthase 1 (ASS1), and ornithine carbamoyltransferase (OTC). The study proposes that liver tumor-initiating cells contribute to the development of T2DM/NASH hepatocarcinogenesis by increasing glucose metabolites and L-arginine levels. This triggers glucose uptake, creatine metabolism, oxidative stress resistance, and tumor cell proliferation [14]. These findings highlighted the association between elevated L-arginine and inhibition of these enzymes.

### 2.3. Formation of Oxidative Stress and Resistance Mechanisms

Various sources contribute to the formation of oxidative stress, including the oxidation of free fatty acids, iron overload, inflammatory cytokines, lipid peroxidation, and mitochondrial dysfunction. Oxidative stress plays a crucial role in the progression of simple steatosis to NASH and hepatocarcinogenesis [56]. When the balance between the production of ROS (such as hydroxyl (OH^•^), superoxide (O_2_^•−^) radicals, and nitric oxide (^•^NO) and non-radical species (H_2_O_2_), as well as the protection from oxidative damage through antioxidant enzyme systems and DNA repair, is disrupted in the liver as in NAFLD/NASH, it can lead to the onset and progression of hepatocarcinogenesis [57]. Excessive ROS production can harm DNA and proteins, disrupt mitochondrial conditions, trigger apoptosis, and alter cellular signaling pathways like MAPK. This can impact regenerative cell proliferation, growth, and angiogenesis [57].

The development of oxidative stress and its influence on hepatocarcinogenesis was studied in NASH animal models. Formation of oxidative stress and damage to liver cell DNA in terms of 8-hydroxy-2-deoxyguanosine (8-OHdG) in TSOD and fatty liver Shionogi (FLS) NASH model mice was considered a key event in the initiation of T2DM/NASH-associated hepatocarcinogenesis [14,58]. From proteomics studies, NASH-associated hepatocarcinogenesis was found to be associated with the activation of nuclear factor (erythroid-derived 2)-like 2 (Nrf2)-mediated signaling, which is known to mediate resistance to oxidative stress injury [13]. In addition, alteration of protein expression of numerous enzymes involved in the control of mitochondrial function was also detected in NASH-associated human HCCs. Human and STAM NASH model mice HCCs exhibited strong overexpression of transcriptional factors prohibitin 1 (PHB1) and prohibitin 2 (PHB2). These factors play crucial roles in controlling mitochondrial respiration, function, cell proliferation, and apoptosis [13]. In addition, the study revealed an increase in the activity of superoxide dismutase manganese [Mn], mitochondrial (SOD2), and thioredoxin (TXN), while catalase (CAT) was found to be decreased in tumors [13]. In another NASH model containing mice fed with a methionine and choline-deficient diet (MCD), proteomic analysis of livers revealed significant elevation of peroxiredoxins 1 (Prdx1) and 6 (Prdx6) in correlation with TGFβ1, TNFα, and TLR4, CYP2E1, cytokeratin 8 (CK8), cytokeratin 18 (CK18), fructose-1,6-bisphosphatase 1 and vimentin, and downregulation of proteins involved in methionine (Met) metabolism and oxidative stress in NAFLD/NASH [27]. From those studies, all observed metabolome and proteome alterations pointed to the formation of oxidative stress resistance in liver tumor cells as a consequence of lipotoxicity and insulin resistance.

Among proteins involved in the development of liver tissue cellular defense against oxidative stress, oxidative DNA damage, inflammation, and steatohepatitis, peroxiredoxins 1 (Prx1) and 6 (Prdx6) and sestrin 2 (SESN2) were considered promising potential diagnostic biomarkers for the early stage of NAFLD/NASH, which may also influence the mTOR [27,28] (Table 1 and Table 2). Prdx1 and Prdx6 are involved in Akt/mTOR, AMPK/mTOR, PI3K/Akt, FoxO and p53 signaling pathways, and ubiquitin-mediated proteolysis [59,60]. It was also demonstrated that protein levels of SESN2 were positively correlated with AMPK/mTOR pathway components and induced expression of lipogenesis-related genes in NAFLD model HFD-fed C57BL/6 mice liver and palmitic acid (PA)-stimulated HepG2 cells [28].

### 2.4. Glycoproteins-Related Pathways in NASH HCC and Potential Biomarkers

Ramachandran et al. have revealed through their study of human NASH-HCC serum samples that cholesterol and fatty acid metabolism regulators, LXR/RXR and FXR/RXR, along with acute phase response signaling, complement system, and clathrin-mediated endocytosis signaling are involved in pathways related to glycoproteins in the development of NASH and HCC [16]. Alpha-2-macroglobulin (A2MG), haptoglobin (HP), apolipoprotein C3 (APOC3), complement factor H (CFAH), serotransferrin (TRFE), vitronectin precursor (VTNC), ceruloplasmin (CERU), and alpha-1 antitrypsin (A1AT) exhibited unidirectional differences in abundance across the three phenotypes. Among the upstream regulators participating in NASH-associated hepatocarcinogenesis, HNF1α, HNF4α, and sterol regulatory element binding factor (SREBF1) were identified as associated with NASH HCC transcription factors.

Zhang et al. found that alpha-1-acid glycoprotein (AGP1) glycopeptides had significantly higher glycan branching, sialylation, and fucosylation in samples from NASH and cirrhosis patients compared to controls. This suggests that AGP-1 glycan could potentially serve as a diagnostic biomarker for NASH and associated HCC [29] (Table 1). In addition, Kamada et al. discovered that fucosylated and hyper-sialylated variants of HP are valuable indicators for distinguishing NASH from NAFLD and HCC from control groups [30] (Table 1). HP glycoforms could be therefore applied as markers for the diagnosis of NASH and associated HCC.

In addition to various glycosylated protein markers, the APOC3 protein exhibits notably decreased levels in NASH HCC, and can be applied for NASH-HCC diagnosis [31] (Table 1). Moreover, specific glycopeptide moieties located at amino acid position 1424 of A2MG are detectable in the plasma of HCC patients [31].

### 2.5. Immune Dysregulation and Activation of Liver Fibrogenesis with the Central Role for TGF-β/SMAD Pathway

In the pathogenesis of NAFLD and NASH, an increase in plasma insulin levels, de novo lipogenesis, TG, and hepatic gluconeogenesis finally resulted in lipotoxicity, damage to hepatocytes, and an influx of immune cells, activation of transforming growth factor β (TGF-β)/SMAD signaling in hepatic stellate cells (HSCs), their differentiation into myofibroblasts, and finally proceeding to progressive fibrosis in the chronic state of NASH [61,62]. It has been shown that stress-related signaling pathways, like c-Jun N-terminal kinase (JNK) and NF-κB, are activated by pro-inflammatory cytokines, such as TNF-α, IL-1, IL-6, STAT3, and ERK. This activation leads to increased hepatocyte proliferation and ultimately promotes the development of hepatocarcinogenesis [61]. HSCs that are not strongly activated generate growth factors and cytokines, including hepatocyte growth factor (HGF), which can help prevent hepatocyte death and the development of HCC [62]. However, in the case of chronic liver injury, a shift to tumor-promoting HSC occurs, giving rise to a highly active HSC with myofibroblastic phenotype with increased deposition of type I collagen and other matrix proteins, which promotes cell proliferation and secreting TGF-β and platelet-derived growth factor (PDGF) [63]. In addition, TGF-β activation boosts the production of TGF-β within the cell and extends the lifespan of activated HSCs by decreasing apoptosis [64]. In addition, integrins, which act as cell surface mechanoreceptors, can interfere with TGF-β1 and PDGF and modulate the proliferation and survival of hepatocytes and HSC [65]. The regenerative repair pathways, such as the Hedgehog signaling pathway, are induced during the process of fibrogenesis in NASH and predict the induction of hepatocarcinogenesis [61].

Besides the hepatocytes, tumor microenvironment stromal cells, endothelial cells, immune cells, cytokines, and extracellular matrix (ECM) have been suggested to play a key role in the initiation and progression of HCC [66,67]. In proteomic studies with human NASH-associated biopsies, in addition to alteration proteins associated with inflammation, lipid deposition, insulin resistance, and oxidative stress, significant elevation of numerous ECM and cytoskeleton proteins, with the majority being downstream of TGF-β, including different collagens, fibronectin, vimentin, beta actin-like protein 2, tropomyosin α-4 chain, myosin 9, tubulin α-1C chain, moesin, and others, have been demonstrated [13,68]. On the contrary, the intermediate filament members CK8 and CK18 were strongly downregulated in human NASH biopsies and HCCs but not in virus-associated HCCs [13,69]. The CK18 fragments in the blood can indicate NASH and the severity of NAFLD in patients with uncontrolled hepatocyte apoptosis, necrosis, and caspase cleavage. A previous study on a diverse group of patients with biopsy-proven NAFLD highlights the potential clinical value of this test [32] (Table 1).

In addition, a large ECM glycoprotein thrombospondin-I (TSP-1) was reported to be a potential target of interest in NAFLD/NASH as it can help to develop anti-fibrotic therapeutics [33] (Table 1 and Table 2). TSP-1 regulates inflammation, cell adhesion, angiogenesis, and several signaling pathways, such as TGF-β1. A comparison of the transcriptomic profiles based on genotype revealed significant differences in the expression of the PPAR pathway and amino acid metabolism pathways in TSP-1 null mice. These findings were further supported by a noticeable decrease in serum lipid levels, indicating activation of the PPAR pathway [33].

The influence of gut bacterial dysbiosis and microbial metabolites in the promotion of NASH hepatocarcinogenesis, worsening liver disease through enterohepatic circulation, has been recently reported [70,71]. It is known that the liver carries out immunological functions and participates in the physiological connection between gut-derived molecules and the systemic circulation, in which liver macrophages, Kupffer cells (KCs) producing pro-inflammatory and pro-fibrogenic cytokines and chemokines, liver resident cells, and monocytes play a very important role [61]. M1 macrophages promote inflammation, whereas M2 macrophages exert inhibitory effects and induce tissue repair. Moreover, pro-inflammatory cytokines, including TNF-α, IL-1β, IL-6, IL-12, C-C chemokine ligand 2 (CCL2) and 5 (CCL5) are secreted by M1 macrophages and exhibit increased amounts of nitric oxide species (NOS) and ROS [61]. Obesity, whether caused by diet or genetics, can affect the gut microbiota and increase the levels of deoxycholic acid (DCA), a metabolite produced by gut bacteria. This can lead to the activation of senescence-associated secretory phenotype (SASP) in hepatic stellate cells (HSCs), resulting in the secretion of inflammatory and tumor-promoting factors (such as IL-1, 6, and 8) in the liver. This process can contribute to the development of hepatocellular carcinoma (HCC) in both human and mouse models [8]. Recent metabolome research has revealed a reduction in serum levels of indole-3-propionic acid, a potential biomarker and a deamination metabolite of tryptophan maintaining intestinal homeostasis, in NASH-HCC patients [12,34] (Table 1).

The use of choline-deficient, L-amino acid-defined, high-fat diet (CDAHFD)-fed NASH model mice in animal experiments revealed severe inflammation, fibrosis, impaired mitochondrial respiration, and severe oxidative stress [72]. However, de novo lipogenesis (DLG), gluconeogenesis, and antioxidant enzymes in the liver were found to decrease. These changes were associated with a reduction in mitochondrial DNA copy number and complex proteins [73]. When exploring NASH biomarkers with gene expression analysis of the whole blood, a marked increase in the inflammation markers such as TNF-α, monocyte chemoattractant protein-1 (MCP1), IL1-β and lipocalin 2 (LCN2) (inflammation), collagen 1a1 (COL1A1) and 3a1 (COL3A1) (fibrosis), and Nrf2 targets NAD(P)H quinone dehydrogenase 1 (NQO1) and heme oxygenase (HO1) was found in the CDAHFD group. On the other hand, CDAHFD induced suppression of stearoyl-coenzyme A desaturase 1 (SCD1), fatty acid synthase (FASN), SREBF-1 (lipogenesis), G6PC and PEPCK (gluconeogenesis), and GPX2, CAT, and SOD3 (antioxidant enzymes) [73]. Recent findings in CDAHFD-treated mice suggested that proteins vitronectin (VN), monoacylglycerol acyltransferase 2 (MGAT2), retinoic acid-inducible gene-I (RIG-I)-like receptor (RLR), and formyl peptide receptor 2 (FPR2) may become potential diagnostic biomarkers and/or promising therapeutic opportunities for NAFLD/NASH by modulating the inflammatory reaction and contributing to the development of fibrosis via the activation of HSCs [35,36,37,38] (Table 1 and Table 2). VN, being an EMT component protein, was shown to increase the COL1a2 mRNA expression levels, decrease CD11b and F4/80, macrophage markers, as well as TNF-α and IL-1β inflammatory cytokines and α-SMA, but did not affect the fatty acid synthases expression [35]. The regulation of TG absorption and homeostasis is crucially dependent on the highly expressed T2 enzyme in both the human small intestine and liver [36]. RLR and FPR2 could be also involved in inflammatory reactions in NASH development [37].

### 2.6. The Role of NRIP1 in NAFLD/NASH and Associated HCC

Recent proteome and bioinformatic analyses of NASH human liver biopsies have revealed a fascinating connection between the development of NAFLD and NASH and the activation of the transcription cofactor NRIP1 (nuclear-receptor-interacting protein 1), also known as RIP140 (receptor-interacting protein, 140 kDa) [13]. NRIP1 is a multifunctional transcriptional co-regulator, which plays important physiological roles in the control of a large number of metabolic nuclear receptors and transcription factors, such as the estrogen-related receptor (ESR1), nuclear receptor subfamily 3 group C member 1 (NR3C1) and member 2 (NR3C2), PPARα, PPARβ/δ, PPARγ, liver-X-receptor (LXRα), retinoid-X-receptor alpha (RXRα), apoAI enhancer, Wnt/β-catenin, RAR related orphan receptor A (RORA), and RORC, and is involved in the regulation of the ARNTL/BMAL1, CLOCK, and CRY1 circadian clock genes expression [74,75,76,77,78]. The NRIP1 gene plays a crucial role in regulating several bodily functions, including energy expenditure, lipid and glucose metabolism, inflammatory response, intestinal homeostasis, ovulation, mammary gland development, behavior, and cognition [74,79,80]. Compared to their wild-type counterparts on an HFD, NRIP1-null mice exhibit remarkable leanness, lack of obesity and hepatic steatosis, improved glucose tolerance, and heightened insulin responsiveness [81]. NRIP1 function as a coactivator for LXR appears to have an impact on its ability to regulate FAS expression in the liver [74,81].

NRIP1 also participates in the regulation of key steps and various oncogenic signaling pathways during cancer initiation and progression, especially in breast, ovary, liver, and colon tumors, and the development of atherosclerosis [79,82,83]. The impact of NRIP1 on HCC has been studied, and findings indicate that decreased levels of NRIP1 (both mRNA and protein) can lead to increased growth and migration of cancer cells in HCC. This is believed to be due to the direct interaction between NRIP1 and β-catenin, leading to repression of β-catenin/T-cell factor/lymphoid enhancer factor (TCF) signaling [78]. Furthermore, in Huh7 and HepG2 human liver cancer cells, overexpression of NRIP1 suppressed the malignant potential of liver cancer cells by inhibiting NF-kappaB (NF-kB)-mediated alternative polarization of macrophages [84], while the downregulation of miR-140-3-p miRNA, which targets the NRIP1 mRNA, was suggested to influence the hepatocarcinogenesis by stimulating anti-apoptotic signaling [85]. In an adrenoleukodystrophy mouse model, the genetic inactivation of NRIP1 prevented mitochondrial depletion and dysfunction, bioenergetic failure, and inflammatory dysregulation. This was achieved through a redox-dependent mechanism driven by very-long-chain fatty acids [86]. From these studies, NRIP1 is implicated in the development of NAFLD/NASH due to metabolic and inflammatory dysregulation and is downregulated in HCC, stimulating anti-apoptotic signaling and acting as an enhancer of proliferation, growth, and malignant potential of liver cancer cells. Further deciphering the link between NRIP1, hormone nuclear receptors, LXR/Fas, PPARs, Wnt/β-catenin/T-cell factor (TCF), and its epigenetic regulation could lead to novel strategies against the development of NASH and associated HCC. Furthermore, the NRIP1 in the form of circulating RNA (circRNA) could become a potential biomarker of NAFLD/NASH-HCC, which needs further investigation (Table 1).

## 3. ER Stress, Mitochondrial Dysfunction, and Inhibition of Autophagy in NAFLD/NASH and Potential Biomarkers

### 3.1. Endoplasmic Reticulum (ER) Stress and Inhibition of Autophagy

The unfolded protein response (UPR) has been closely linked to NAFLD [47,87]. There is still uncertainty regarding whether ER dysfunction and UPR activation come before hepatic steatosis, leading to NAFLD/NASH, or if these processes are a result of lipogenesis activation in hepatocytes [88,89,90,91]. The lumen of the ER is responsible for producing both membrane and secretory proteins. Meanwhile, the UPR pathway serves as a valuable tool for monitoring protein stress levels and ensuring ER homeostasis is maintained. Under normal conditions in an inactive state, the 78-kDa glucose-regulated protein (grp78) could bind to UPR sensors (protein kinase R-like ER kinase (PERK), activating transcription factor 6 (ATF6) and inositol-requiring enzyme 1 (IRE1)). When there is an increase in unfolded proteins, it triggers the dissociation of grp78 from UPR sensors during ER stress. This then activates the UPR by promoting PERK dimerization, facilitating ATF6 translocation to the Golgi apparatus, and inducing IRE1 autophosphorylation. A close relationship was found between ER stress and autophagy processes [92,93]. In the ER, autophagy plays a crucial role in degrading mutant proteins in alpha-1 antitrypsin deficiency. However, in cases of obesity and alcoholic liver disease, the impaired function of autophagy leads to the formation of damaging Mallory-Denk bodies and ultimately, cell death. The deficient autophagy contributed to liver steatosis, endoplasmic reticulum stress, and progression of liver disease [94].

Autophagy is a dynamic process which bound to lipid metabolism by degrading metabolic waste and accumulated toxic endogenous substances, protein aggregates/condensates, and even whole organelles such as mitochondria, ER, peroxisomes, lysosomes, and lipid droplets [92,93]. Suppression of autophagy could induce toxicity and affect cell survival and hepatocarcinogenicity in NAFLD/NASH [92] (Figure 2). It is influenced by various factors, including changes in nutritional status, such as feeding or fasting. In addition to the well-known mTOR/AMP-activated protein kinase (AMPK) pathways, other factors, such as phosphoinositide interacting 2, sirtuin 1, and perilipin 5, play important roles in regulating autophagy. Perilipin 5, for example, helps to maintain the balance between lipolysis and lipogenesis. Hormones like fibroblast growth factor (FGF21) and protein kinase A-Jumonji domain-containing protein D3, as well as lysosomal enzymes like cathepsins B and L, also affect autophagy. Overall, the regulation of autophagy is a complex process that involves multiple pathways and factors [95].

The suggested mechanism of autophagy regulation by mTOR was the influence towards integration of nutrient signals and growth factors through the phosphorylation of Ser757 and blocking the Unc-51-like autophagy activating kinase 1 (ULK1) activation and its interaction with AMPK [96,97]. Furthermore, the mechanisms underlying HFD-induced NAFLD in mice and its upregulation were related to the alleviation of the lipid peroxidation and ferroptosis and suppression of autophagosome biogenesis through AMPK- and AKT-mediated mTOR activation, decreasing ATG7, Nrf2, and stabilizing KEAP1 [98]. The AMPK/mTOR-mediated autophagy was proposed as a promising approach in NAFLD treatment [96,98].

In the NAFLD/NASH, the involvement of impaired autophagy in the development of liver oxidative stress [99]. Nrf2 plays a critical role in not only safeguarding against oxidative damage via the KEAP1-Nrf2 pathway but also in promoting autophagy and serving as a vital factor in the development of NAFLD/NASH hepatocarcinogenesis. This is due to its ability to enable the expression of pro-survival genes and protect HCC-initiating cells from oxidative stress-induced death [61].

### 3.2. Biomarkers and Potential Molecular Targets Associated with ER Stress and Autophagy in NAFLD/NASH

Several potential biomarkers of NAFLD/NASH were investigated in relation to autophagy and ER stress. In a recent study, Choi et al. performed targeting a novel adipokine gremlin-1 (GR1) and discussed its role in ER stress and autophagy [39]. It was demonstrated that heightened GR1 levels can cause insulin resistance in skeletal muscle, adipocytes, and hepatocytes. Additionally, they can result in increased lipid accumulation, lipogenesis, ER stress markers, EGFR expression, mTOR phosphorylation, and reduced autophagy markers in cultured primary hepatocytes [39]. The potential benefits of GR1 as a predictive diagnostic marker and therapeutic method for metabolic diseases, such as NAFLD and hepatic steatosis in obese individuals, are linked to its ability to induce hepatic ER stress by hindering autophagy [39] (Table 1 and Table 2).

Hepatitis B virus x protein (HBx), a protein from hepatitis B virus, has been identified as a risk factor for NASH in relation to ER stress. This is due to its ability to activate mTOR and promote cholesterol transport from the lysosome to the ER through Niemann-Pick type C1 (NPC1)/oxysterol-binding protein-related protein 5 (ORP5) [40] (Table 1 and Table 2). On the basis of metabolomic and transcriptomic screens and public database analysis, Lan et al. have revealed that M1-type polarization of macrophages induced by ER stress was associated with the HBx-related hepatic NASH phenotype. Lan et al. discovered that ER stress triggered by ER oxidoreductase-1-like protein alpha (ERO1alpha) led to M1-type polarization of macrophages, which was linked to the HBx-related hepatic NASH phenotype. This conclusion was drawn from metabolomic and transcriptomic screens as well as public database analysis. HBx-associated NASH immune injury may be predicted through macrophage polarization and dysregulation of cholesterol homeostasis, which could serve as potential biomarkers. Furthermore, reduced levels of high-density lipoprotein cholesterol (HDL-C) and an elevated monocyte-to-HDL-C ratio (MHR) were significantly associated with mortality in HBV patients, suggesting their potential use as prognostic biomarkers. HBx-associated NASH immune injury may be predicted through macrophage polarization and dysregulation of cholesterol homeostasis, which could serve as potential biomarkers. In addition, reduced levels of high-density lipoprotein cholesterol (HDL-C) and an elevated monocyte-to-HDL-C ratio (MHR) were significantly associated with mortality in HBV patients, suggesting their potential use as prognostic biomarkers [40]. In addition, leucine aminopeptidase 3 (LAP3) plays a crucial role in autophagy and aids in the degradation of proteins and metabolism of peptides by removing the amino acid leucine. It has been identified as a potential diagnostic biomarker for NASH in the serum of patients with chronic HBV and NASH [41] (Table 1 and Table 2).

Liu et al. applied the combination analysis of metabolomics and proteomics of liver tissues firstly to discover the candidate targets and potential molecular pathways involved in hydrogen sulfide (H2S) mitigating the NAFLD. Their results indicated that perilipin 2 (PLIN2) may become a potential target of hydrogen sulfide (H2S) via the regulation of lipid droplet degradation in alleviating NAFLD [42] (Table 1 and Table 2). In another study, Yao et al. reported that carnitine palmitoyltransferase-2 (CPT-2) dysfunction and inactivity could be the key event in the novel pathogenesis for NAFLD/NASH in the hepatocarcinogenesis pathway and its potential to become a potential therapeutic target of this disease [43] (Table 1 and Table 2). CPT-2 regulates long chain fatty acid β-oxidation on the inner mitochondrial membrane (IMM) and could be involved in Wnt/β-catenin signaling.

#### 3.2.1. p62

The multifunctional protein SQSTM1 (p62) plays a crucial role in various biological processes, such as metabolism, inflammation, oxidative stress resistance, ER stress, apoptosis, and autophagy. It is unique in its involvement in ubiquitin-related autophagy, as well as Nrf2 and mTOR signaling. The activation of mTORC1, Nrf2, and NF-kB is dependent on its presence [44,100]. The multifunctional protein SQSTM1 (p62) is essential to promote Rag protein dimerization and affect TRAF6 for mTORC1 and NF-kB activation, respectively. Accumulation of p62 was detected in premalignant and cancerous liver diseases, including HCC [45]. The involvement of p62 in the development of liver tumors has been established, and it has been found that increased levels of p62 in non-tumor liver tissue can serve as a reliable biomarker for predicting the recurrence of HCC following therapeutic ablation, as demonstrated in both human and mouse models [44] (Table 1 and Table 2). Increased p62 protein levels were found in the livers of NASH model mice with a combination of genetic and nutritional factors, namely, C57BL/6 mice fed MCD or high fat–high cholesterol (HFHC) diet, *db/db* mice fed MCD, and *foz/foz* mice administered HFD [46]. Furthermore, serum p62 exhibited a high overall accuracy in discriminating patients with NAFLD from control subjects and was reported as a potential non-invasive biomarker and an independent risk factor for the clinical diagnosis of NAFLD and NASH [46]. The transcriptomics analysis revealed that p62 was overexpressed in the livers of NASH patients and C57BL/6NJ mice with NAFLD/NASH who were given Western HFD and sugar water. This led to an increase in the expression of genes that are known to be involved in inflammation, fibrogenesis, and metabolism, all of which are altered in NASH and NASH-HCC [101]. The role of p62 in selective autophagy, working together with wild-type Huntingtin, a scaffold protein that interacts with ULK1 during autophagy initiation, is believed to be highly relevant to the development of NAFLD and NASH [45,46].

#### 3.2.2. CNPY2

Canopy homolog 2 (CNPY2) is an interesting protein and angiogenic growth factor which is present in ER lumen, participating in ER stress, and is likely to play a role in NASH-associated liver cancer progression [47,48]. This molecular target shows promise in addressing hepatocarcinogenesis and serves as a valuable prognostic indicator for various neoplasms [49]. This protein is found in several areas within cells, including the ER lumen, plasma membrane, intracellular space, and perinuclear region [102]. Several studies have demonstrated its role in embryogenesis by promoting the growth of neurites through increased phosphorylation of myosin regulatory light chain (MRLC) [103], a crucial mechanism in the regulation of myosin activity and cell motility. Furthermore, it was demonstrated that CNPY2 can improve the spread of fibroblast cells and the migration of rat C6 glioma cells. CNPY2 has also been identified as playing a crucial role in ER stress and UPR-related diseases, including metabolic disorders, NAFLD, inflammation, and cancer, by regulating the PERK-C/EBP homologous protein (CHOP) pathway [47,87] (Table 1 and Table 2). The study revealed the presence of Mcp1, Il1-β, and Lcn2 (markers of inflammation), as well as Col1a1 and Col3a1 (markers of fibrosis), and Nqo1 and Ho1 (Nrf2 target genes). Consequently, the enzymatic activity of PERK kinase increases, leading to a cascade of events, including induction of CHOP. The deletion of CNPY2 has been shown to block the PERK–CHOP pathway, and this protected against NAFLD induced in animal models by HFD or with tunicamycin [47].

Upon ER stress, CNPY2 is released from grp78 and upregulated through CHOP signaling at the transcriptional level [47]. It was found to induce ER stress and unfolded protein response in HepG2 and Huh7 cells. This effect was dependent on its ability to suppress proteolysis and stabilize actomyosin complexes, which could contribute to the invasion and metastasis of tumor cells [48]. Proteome analysis of CNPY2 null HepG2 and Huh7 cells demonstrated the downregulation of numerous ER stress-related proteins. An important role of CNPY2 in cell survival proliferation and invasion potential has been confirmed in experiments with human liver cancer cells [48,104]. Furthermore, an association of CNPY2 and induction of cell proliferation was demonstrated in the liver CNPY2-positive altered foci, HCAs, and HCCs in relation with Nrf2 activation [50]. The function of CNPY2 and its capacity to become the biomarker of NASH and associated HCC should be examined in future investigations.

#### 3.2.3. CACHD1

The novel protein, CACHD1, was identified through proteome analysis in the HCCs of 18-week-old mice in the STAM mouse NASH model. Its overexpression was confirmed in CACHD1-positive foci, adenomas, and carcinomas through immunohistochemical analysis of the livers of 10- and 18-week-old STAM mice. These mice exhibited increased hyperglycemia, hepatic lipid accumulation, oxidative stress, impaired insulin secretion, and insulin resistance [50,105]. CACHD1 is a newly discovered protein that interacts with Wnt-receptors, acts as a potential calcium channel and chemotaxis receptor, and is elevated in the male mammalian central nervous system. It has been found to play important roles in regulating neurogenesis, neuronal identity, Notch signaling, and Alzheimer’s disease [106,107,108,109].

The involvement of CACHD1 in regulating protein folding, unfolded protein response, autophagy, apoptosis, and cytoskeleton organization was demonstrated through proteome analysis of Huh7 and HepG2 cells with CACHD1 knockdown, as well as in vivo immunohistochemical analyses [50]. The expression of CACHD1 in tumors and altered foci was found to be correlated with an overexpression of the phosphorylated form of protein kinase R-like endoplasmic reticulum kinase (P-PERK). As CACHD1 plays a crucial role in regulating the cell cycle and autophagy process, it has been identified as a potential early biomarker of liver preneoplastic and neoplastic lesions associated with NASH (Table 1 and Table 2). Additionally, CACHD1 could serve as a potential target protein in diabetes/NASH-associated hepatocarcinogenesis [50].

### 3.3. Methylation and Epigenetic Changes

Activation of methylation was found in TSOD and STAM NASH model mice HCCs via metabolome and methylation analyses [14,110]. The S-adenosylmethionine (SAM)/S-adenosyl-L-homocysteine (SAH) ratio elevation indicates a disruption in regulating SAM synthesis and catabolism. This disruption can cause SAM accumulation in the liver of mice, potentially leading to the development of NAFLD and HCC [111]. The formation of m5dC in DNA, triggered by methyl radical mediation, is believed to be the cause of the increased incidence of liver tumors in STAM mice. Furthermore, the inflammation observed in NASH STAM mice livers may be linked to the production of methyl radicals [110]. SAM biosynthesis in the liver is controlled by methionine adenosyltransferase 1A (MAT1A) [51]. By conducting a phospho-proteomics analysis on the livers of MAT1A knockout mice, it was discovered that SAM treatment may play a role in liver pathology. Specifically, the phosphorylation of La-related protein (LARP1), which is sensitive to SAM, was found to be a potential factor in the progression from NASH to HCC. This finding was further supported by an increase in LARP1 expression in human NASH and HCC, suggesting its potential use as a biomarker for NASH hepatocarcinogenesis [51].

## 4. New Therapeutic Approaches and Molecular Targets in NAFLD/NASH and HCC

The importance of target pathways in NAFLD/NASH without inhibiting the critical roles in normal liver cells is pointed out for NAFLD/NASH prevention and effective therapies [19]. All therapeutic studies help investigate the potential molecular targets of NAFLD/NASH-HCC to alleviate harmful effects; however, to find more specific targets, novel approaches are necessary for the determination of the concrete role of each component and evaluation of non-discovered molecules in the development of NAFLD/NASH and associated HCC. In addition, recent studies have also discussed the beneficial effects of bariatric surgery on fibrogenesis and the incidence of NASH and associated HCC [112,113].

### 4.1. AMPK/mTOR Pathway-Associated Targets and Therapeutic Agents

Targeting the mTOR signaling pathway might be a novel therapeutic strategy of T2DM-associated NALFD. It was proposed that aging-related factors, such as mTOR, nicotinamide adenine dinucleotide (NAD(+)), and silence information regulator protein family (sirtuins), play pivotal roles in the development of NAFLD-associated HCC and other age-related liver diseases [114,115,116].

It has been recently reported that natural products, including phenols, flavonoids, glycosides, quinones, terpenoids, pyrazines, alkaloids, and phenylpropanoids, have antioxidant, energy-producing, anti-inflammatory, anti-apoptotic, and anti-aging effects, which mainly influence the AMPK/Nrf2/mTOR, Nrf2/HO-1, NAMPT/NAD(+)/SIRT, AMPK/SIRT1/PGC-1alpha and PKCs/PARPs/NF-kB signaling pathways, thereby regulating NAD(+) metabolism to prevent and treat NAFLD [117]. Hepatoprotective effects, caused by the inhibition of PI3K/Akt/mTOR signaling and effective alleviation of oxidative stress damage and ER stress induced by excessive lipid accumulation in hepatocytes, and activation of autophagy were observed for agonists of AMPK thymoquinone [118] and 5-aminoimidazole-4-carboxamide-1-beta-D-ribofuranoside (AICAR) [119], agonist of GLP-1/glucagon cotadutide [120], an antagonist of sphingosine 1-phosphate receptor 2 (S1PR2) JTE-013 [26], activator of PI3K, autophagy-related genes (ATG) and agonist of Nrf2, PPARα and hemeoxygenase-1 (HO-1) pterostilbene [121], flavonoid with antioxidant properties involving CYP7A1, quercetin [122], Pueraria flavonoids [92], a novel gerotherapy compound, BIOIO-1001 [123], a natural nonenzymatic antioxidant with low toxicity schisandrin B (Sch B) [124], H1-antihistamines (AHs) [125], fat soluble polyphenol Tanshinone IIA (TsIIA) [126], purendan (PRD) [127], down-regulating the expression of p-mTOR, p-S6K1 and SREBP-1c, chrysin [128], red pepper seed extract (RPS) [129], Gardenia Fructus (GF) extracts [130], curcumin [131], and magnesium [132].

In a recent study, a dual inhibitor of soluble epoxide hydrolase (sEH) and cyclooxygenase 2 (COX-2), 4-(5-phenyl-3-{3-[3-(4-trifluoromethylphenyl)-ureido]-propyl}-pyrazol-1-yl)-benzenesulfonamide (PTUPB), significantly reduced the expression of liver senescence-related molecules p16, p53, and p21 in HFD mice and inhibited the PI3K/AKT/mTOR pathway through Sirt1 [130]. PTUPB improved autophagy, slowed down the senescence of hepatocytes, and alleviated NAFLD in vitro [133].

Protein ghrelin was found to be widely distributed in peripheral tissues and the central nervous system and plays a vital role in regulating food intake, energy balance, and substance metabolism. The blockade of central ghrelin receptor can treat NAFLD via the hypothalamic PI3K/Akt/mTOR signaling pathway to improve insulin resistance [134].

In addition, the mitochondrial pyruvate carrier (MPC) has emerged as a therapeutic target in T2DM and NASH due to its activation of the AMPK/mTOR axis [135]. Yano et al. demonstrated that fibroblast growth factor 21 (FGF21), an endocrine growth factor, induced the phosphorylation of AMPK at Thr172 and Raptor at ser792 and suppressed mTOR at ser2448, which downregulated mTORC1 signaling and contributed to improved liver steatosis in HFD-fed mice [136]. In addition, overexpression of follistatin, (FST), a gonadal protein that specifically inhibits follicle-stimulating hormone release, alleviated lipid accumulation and lipogenesis, whereas FST knockdown aggravated hepatic steatosis via suppressing the mTOR pathway, therefore, may have a potential to become a therapeutic target in NAFLD/NASH [137]. Very recently Chen et al. have shown that Ufm1-specific ligase 1 (Ufl1) and Ufm1-binding protein 1 (Ufbp1), which are putative targets of ubiquitin-fold modifier 1 (Ufm1), prevent liver fibrosis, subsequent steatohepatitis, and HCC development in diethylnitrosamine DEN-treated mice by inhibiting the mTOR pathway [138].

### 4.2. Lysosome Targetting

A potential molecular target participating in lipid homeostasis and hepatic inflammation is the Odd-skipped related 1 (Osr1) gene, which plays a critical role in embryonic development as a cancer repressor gene. It has been recently reported that Osr1 disruption contributes to NAFLD progression [139]. It was recently found that Osr1 heterozygous mice fed HFD for 10 weeks, regardless of sex, exhibited more severe steatosis in the liver compared to wild-type mice. In addition, Osr1 stimulated upregulation of lipogenesis protein, including SREBP-1c, overactivated Akt/mTOR, JNK, and NF-kB signaling, impaired IRS2 expression, and depressed hepatic expression of Cyp7a1 and Cyp27a1 and bile acid synthesis [139].

The multiple functions of the lysosome, including degradation, nutrient sensing, signaling, and gene regulation, enable the lysosome to regulate lipid metabolism in NAFLD. Therefore, targeting the lysosome has been proposed as a promising strategy for manipulating lysosome functions and autophagy in NAFLD [140]. Transcription factors TFEB and TFE3 that are negatively regulated by mTORC1 also primarily regulate the autophagy process. The transcription factor EB (TFEB) was reported as a potential regulator of lysosomal function and autophagy and attracted attention as a therapeutic target [141]. Furthermore, mTOR-mediated phosphorylation on TFEB regulates its subcellular localization and activity at the lysosomal surface and plays a critical role in physiological processes, such as lipid metabolism, and the dysfunction of TFEB has been observed in the pathogenesis of several diseases, including NAFLD [141]. Gong et al. demonstrated that elevated levels of TFEB alleviated the development of NAFLD, possibly through the upregulation of fibroblast growth factor 21 (FGF21) expression by targeting the promoter of FGF21 [142].

Folliculin (FLCN), a negative regulator of TFEB and TFE3 and an activator of mTORC1 due to its GAP activity towards RagC/D, was suggested as one of the beneficial molecular targets in NAFLD/NASH. Its loss resulted in the alleviation of NAFLD/NASH (e.g., reduced triglyceride accumulation, fibrosis, and inflammation) in Flcn knockout mice exposed to a NASH-inducing diet [143]. Furthermore, FLCN was shown to specifically activate the autophagy and lysosomal biogenesis while leaving mRNA translation machinery unperturbed, therefore, opening new possibilities for treatment strategies through its role in NAFLD and NASH by controlling hepatocyte homeostasis. It was proposed that targeting FLCN could avoid inhibiting targets of the canonical mTORC1 pathway, including p70S6K, which can both stimulate and inhibit DNL [19].

### 4.3. Natural Killer T (NKT) Cells as New NAFLD/NASH Targets

Recently, transcriptome analysis of the human liver demonstrated aberrant cholesterol metabolism and natural killer T (NKT) cell dysfunction in NAFLD patients [144]. Furthermore, the hepatic cholesterol accumulation in obesity has been proposed to selectively suppress natural killer T (NKT) cell-mediated antitumor immunosurveillance in high-fat high-carbohydrate diet-associated orthotopic and spontaneous NAFLD-HCC mouse models. mTORC1/SREBP2/cholesterol-mediated NKT dysfunction in the tumor-promoting NAFLD was proposed as one of the intervention strategies targeting NKT cells to control HCC development in NAFLD/NASH. Treatment with Rosuvastatin and mTOR inhibitor Vistusertib was reported to restore NKT expansion suppressed hepatic cholesterol biosynthesis and prevented obesogenic diet-promoted HCC development [144].

### 4.4. Non-Coding RNAs as Biomarkers and Therapeutic Agents

Non-coding RNAs (ncRNAs) were found to play an important role in both biological processes and disease pathogenesis, and microRNAs that can target and regulate the expression of both mTOR and SREBP1c were discovered. Wang et al. have observed miR-30a-3p ncRNA to be substantially elevated in the blood of NAFLD patients and elucidated its functions and biomarkers and potential targets of NAFLD/NASH-HCC in vitro using HepG2 cells [145]. Bioinformatics analyses showed that PPAR-alpha was a possible target of miR-30a-3p which suggested that miR-30a-3p/PPAR-alpha may be developed as a potential agent in NAFLD, which is enough to attenuate TG accumulation and hepatic steatosis [145]. Recently, the impact of ncRNAs miR-615-5p (short) and H19 (long), which target the mTOR, and act in the mTOR/SREBP1c axis, exert their functional impact in suppressing the accumulation of lipid droplets and TG in Huh-7 cells was reported [146]. In another study, ncRNA miR-122-5p was found to attenuate inflammatory response and oxidative stress damage in dietary-induced NAFLD possibly via the upregulation of mTOR downstream FOXO3 in palmitic acid (PA)-treated L02 cells and HFD-induced fatty liver [147]. Liu et al. have further identified the long ncRNA (lncRNA) SNHG6 as an enhancer of cholesterol-dependent mTORC1 lysosomal recruitment and activator of FAF2-mTOR interaction at ER-lysosome contacts, which increased progression from NAFLD to HCC [148]. In addition, other long lncRNAs (lnc027912, Smarca2, Tacc1, Flywch1, and Mef2c) are involved in the regulation of the lipid accumulation, and inflammatory and metabolic pathways, such as mTOR/AMPK/SREBP1c signaling in NAFLD [149,150]. In addition to these results, Xu et al. recently demonstrated by applying the bioinformatic analysis that PTEN-induced putative kinase 1 (PINK1)-mediated mitophagy is inhibited in NASH mouse liver with fibrosis via the axis of upstream circular RNA 608 (circ608)-microRNA 222 (miR222)-PINK1 (circ608 inhibiting miR222) through mTOR and Wnt pathways [151]. Moreover, another potential therapeutic target, miR-149, was shown to suppress the progression of NAFLD in an HFD-treated mouse model by downregulating the expression of inflammatory factors (TNF-alpha, IL-1beta, IL-6, and NF-kB) and apoptotic-related factors (caspase-12 and CHOP), thus, alleviating the ER stress-induced inflammation and apoptosis by inhibiting the activating transcription factor 6 α (ATF6) signaling pathway [152]. The impact of ncRNAs as potential therapeutic targets in the management of NAFLD/NASH-HCC should be examined in further investigations.

## 5. Conclusions

In the present review, we summarized the recent information on mainstream molecular pathways and novel potential biomarkers/targets in NAFLD/NASH and associated HCC. The mTOR pathway is the foremost multifunctional orchestrator of NAFLD/NASH progression and associated HCC development responding to metabolic syndrome, diabetes, hyperinsulinemia, insulin resistance, and dyslipidemia. The application of inhibitors of mTOR and other regulators implicated in NAFLD/NASH helped us better understand its orchestrating role and further allowed the discovery of novel potential biomarkers in NAFLD/NASH and HCC. The transcriptomics, proteomics, metabolomics, and lipidomics analyses will allow the comparison of the altered gene, protein, metabolite, and lipid expression profiles to emphasize the functional importance of each pathway, reveal their crosstalk, and give a possibility of a complex view on this “multihit” disease progression. A better understanding of these complex pathways will open the door to the discovery of novel targets for drug development. The multi-targeted approach could be used to treat this complex disease and to prevent the resistance upon activation of compensatory pathways in cancer cells. More studies are needed to investigate novel potential mTOR, oxidative stress, ER stress and autophagy, glycoproteins, and epigenetics-related biomarkers/molecular targets application in clinical practice. In addition, the development of new multi-therapy inhibitors in targeted therapies and the role of novel natural compounds in the treatment of NAFLD/NASH appeared to be of great importance in fighting NAFLD/NASH and associated liver cancer.

## Figures and Tables

**Table 1:** NAFLD/NASH/NASH-HCC potential biomarkers, therapeutic targets, and altered pathways.

Gene/Protein/Metabolite	Altered Pathway	Model, Function, Type	Paper
G protein-coupled receptor 180 (Gpr180)	mTORC1, TGF-β, SREBP1, SREBP2	NAFLD diagnosis, therapeutic target. HFD-treated Gpr180KO mice. Huh7 cells. Impact lipid metabolism and cholesterol homeostasis.	[24]
Stimulator of interferon response cGAMP interactor 1 (STING1)	MTORC1, NF-kB	NAFLD/NASH diagnosis, therapeutic target. Biopsy-proven NAFLD patient’s liver, HEK293T (GNHu17), and Hep3B cells. Palmitic acid-induced MTORC1 activation. Increased STING expression was observed in Kupffer cells in patients with NASH.	[25]
Sphingosine 1-phosphate receptor 2 (S1PR2)	PI3K/AKT/mTOR, JAK/STAT, NF-kB, ERK	Prognostic biomarker for human NAFLD/NASH-HCC. Regulates key cellular processes, including the G1/G2 phase of the cell cycle, proliferation, invasion, migration, and apoptosis in Huh 7 and HepG2 cells. Responsible for progression from NAFLD to NASH.	[26]
Arginase 1 (ARG1)	mTOR	Prognostic marker of human metabolic syndrome/T2DM-associated HCC (downregulated). Arginine metabolism (urea cycle) shifts to phosphocreatine.	[14]
Leucine (Leu)	mTOR	Non-invasive diagnosis of NASH HCC. NASH-HCC patients (depletion). Apoptosis of liver cancer cells.	[12]
Arginine (Arg)	mTOR	T2DM-associated NASH-HCC diagnosis. TSOD mice HCC (elevation)	[14]
Peroxiredoxin 1 (Prdx1) Peroxiredoxin 6 (Prdx6)	Akt/mTOR, AMPK/mTOR, PI3K/Akt, FoxO, p53, TGFβ1, TNFα	The early stage of NAFLD diagnosis. Cellular defense against oxidative stress, oxidative DNA damage, inflammation, and steatohepatitis. Ubiquitin-mediated proteolysis.	[27]
Sestrin 2 (SESN2)	AMPK-mTOR	The early stage of NAFLD/NASH diagnosis. NAFLD in vitro model (HepG2 cells) treated with palmitic acid; C57BL/6 mice fed HFD.	[28]
Alpha-1-acid glycoprotein 1 (AGP-1) glycan	JAK2-STAT3	NASH and NASH-HCC diagnosis (human). Can be aided by monitoring changes in sialylation and fucosylation of AGP in the serum may serve as new biomarkers for HCC and cirrhosis.	[29]
Haptoglobin (HP)	IL6, CEBPB, TLR4	NAFLD/NASH diagnosis. Helps to distinguish NASH and NAFLD patients. Fucosylation abnormalities of HP are closely related to HCC. Antioxidant and iron metabolism pathways.	[30]
Apolipoprotein C3 (APOC3)	TLR2/NF-κB	NASH-HCC diagnosis (downregulation). Glycosylated protein marker. Raises plasma TG through inhibition of LPL and stimulation of VLDL secretion and is a novel factor in modulating intestinal triglyceride trafficking. Polymorphism in APOC3 gene might be a risk factor for NAFLD among Asians.	[31]
Cytokeratin 18 (CK18) fragment	TGF-β1, TNF	Increase in CK18 fragments in the blood indicates NASH and the severity of NAFLD in patients with uncontrolled hepatocyte apoptosis, necrosis, and caspase cleavage. Strongly downregulated in human NASH biopsies and HCCs.	[32]
Thrombospondin-I (TSP-1)	TGF-β1, PPARα	Potential therapeutic antifibrotic target in NAFLD/NASH. CDAHFD-fed TSP-1 deficient mice. The critical modulator in NASH.	[33]
Indole-3-propionic acid	AhR, PXR	NASH-HCC diagnosis. Gut bacteria-produced tryptophan metabolite and potential serum biomarker and a metabolite maintaining intestinal homeostasis in NASH-HCC patients.	[34]
Vitronectin (VN)	TGF-β1	NASH and associated HCC diagnosis. CDAHFD-treated VNKO mice. EMT component protein promotes fibrosis and NASH (to distinguish from simple steatosis).	[35]
Monoacylglycerol acyltransferase 2 (MGAT2)	SIRT1, XBP1	NASH and HCC diagnosis, therapeutic target. Murine NASH models and NASH-HCC patients. Highly expressed in the human small intestine and liver. Regulates TG absorption and homeostasis. Modulates inflammatory reaction and contributes to the development of fibrosis.	[36]
Retinoic acid-inducible gene-I (RIG-I)-like receptor (RLR)	NFkB	NAFLD/NASH diagnosis and potential therapeutic target. Modulates the inflammatory reaction and contributes to the development of fibrosis via activation of HSCs. Promotes fibrosis and NASH.	[37]
Formyl peptide receptor 2 (FPR2)	TNF	NAFLD/NASH diagnosis and potential therapeutic target. Modulates the inflammatory reaction and contributes to the development of fibrosis via activation of HSCs.	[38]
Nuclear receptor-interacting protein (NRIP1)	TNF, LXR/Fas, PPARs, Wnt/β-catenin/TCF	NAFLD/NASH/HCC diagnosis and molecular therapeutic target. Regulates energy expenditure, lipid, and glucose metabolism, inflammatory response, intestinal homeostasis, ovulation, mammary gland development, behavior, and cognition functions.	[13]
Gremlin-1 (GR1)	mTOR	NAFLD/NASH predictive diagnostic marker and a potential therapeutic target. Cultured primary mouse hepatocytes, HFD-fed mice. GR1 was suggested to promote ER stress in the liver due to the impairment of autophagy.	[39]
NPC1/oxysterol-binding protein-related protein 5 (ORP5)	mTOR	NASH diagnosis and potential target in hepatitis B virus x protein (HBx)-associated patients. Cholesterol transport from the lysosome to the ER.	[40]
Leucine aminopeptidase 3 (LAP3)	p38, ERK, autophagy pathways	NASH diagnosis (serum biomarker). Chronic hepatitis B (CHB) patients combined with NASH (CHB + NASH).	[41]
Perilipin 2 (PLIN2)	Insulin, PPARγ, TNF	NAFLD diagnosis, molecular target. Regulation of lipid droplet degradation.	[42]
Carnitine palmitoyltransferase-2 (CPT-2)	Wnt/β-catenin, INSR, PPARα, TNF	NAFLD/NASH potential target. Regulates long-chain fatty acid β-oxidation on the inner mitochondrial membrane (IMM). Liver lipid accumulation.	[43]
Sequestosome 1 (SQSTM1, p62)	mTOR, Nrf2, NF-kB	Predictive biomarker of NAFLD, NASH and HCC recurrence/therapeutic target. Human, HFD-SW-treated mice. Autophagy, antioxidant defense.	[44,45,46]
Canopy homolog 2 (CNPY2)	PERK–CHOP	NASH HCC diagnosis, and potential therapeutic target. HCC patients, HepG2 and Huh7 cells. CNPY2 plays a role in ER stress and UPR-related diseases (e.g., metabolic disorders, NAFLD, inflammation, and cancer).	[47,48,49]
Cache domain-containing 1 (CACHD1)	PERK, Wnt, Notch	NASH-HCC diagnosis. STAM mice NASH model, Huh7, and HepG2. CACHD1 is involved in the regulation of protein folding, unfolded protein response, autophagy, apoptosis, and cytoskeleton organization	[50]
La-related protein 1 (LARP1)	SAM-sensitive mechanism	NASH-HCC diagnosis. LARP1 expression was increased in human NASH and HCC.	[51]

Abbreviations: TLR2, Tall-like receptor 2; SIRT1: sirtuin 1; AhR, aryl hydrocarbon receptor; XBP1, X-box binding protein 1, FoxO, Forkhead box transcription factor; PXR, pregnane X receptor; CCAAT enhancer binding protein beta, INSR, insulin receptor.

**Table 2:** Recently discovered NAFLD/NASH/NASH-HCC biomarkers and molecular targets.

Marker	Diagnosis (D)/ Prognosis (P)/Target (T)	Function/Role in Cell	Subcellular Localization
Gpr180	NAFLD (D)	Regulation of vascular remodeling, lipid metabolism, and cholesterol homeostasis.	C
STING1	NAFLD/NASH (D)	Transmembrane protein that functions as a major regulator of the innate immune response, activation of MTORC1, detection of cytosolic nucleic acids.	AV, ER, GA, M, P, N
S1PR2	NAFLD/ NASH-HCC (P)	Biologically active lysophospholipid, actin cytoskeleton reorganization, G-protein coupled receptor signaling pathway, positive regulation of cell proliferation.	CSF, CM, C, PM
ARG1	NASH-HCC (P)	Arginine catabolism, adaptive immune response, aging, cellular response to hydrogen peroxide, transforming growth factor beta stimulus.	AG, C, ES, MOM
SESN2	NAFLD/NASH (D)	Regulation of macroautophagy, gluconeogenesis, growth, apoptosis, signaling, mitochondrial cell death, oxidative stress resistance.	C, N
TSP-1	NAFLD/NASH (T)	Migration, proliferation, chemotaxis, apoptosis, adhesion, cell spreading.	CSF, C, ER, Exo, EM, ES, N, PM, SG
VN	NASH/NASH-HCC (D)	Fibrogenesis, migration, adhesion, cell spreading, proliferation, invasion.	CSF, C, DRLRF, ER, EM, ES, GA
MGAT2	NASH/NASH-HCC (D)	ER stress response, differentiation, quantity, number, storage in, dipeptide repeat protein sensitivity.	C, GA, R
RLR	NAFLD/NASH (D, T)	Modulation of inflammatory reaction and development of fibrosis, L-12 Signaling and production in macrophages, enzyme regulator activity, elongation.	C
FPR2	NAFLD/NASH (D, T)	Modulation of inflammatory reaction and development of fibrosis, chemotaxis, cellular infiltration by, adhesion, migration, signaling receptor activity.	CSF, CM, C, PM, SG
GR1	NAFLD/NASH (D, T)	ER stress, autophagy, proliferation, differentiation, growth, morphogenesis, apoptosis, migration, chemotaxis.	CSF, C, ES
ORP5	NASH (D, T)	ER stress, autophagy, cholesterol metabolic process, cholesterol transport, Golgi to plasma membrane transport, lipid transport, phospholipid transport, lipid transporter.	C, ER, IMBO
LAP3	NASH (D)	Autophagy, proliferation, degradation of proteins, metabolism of peptides by removing the amino acid leucine.	C, M, Exo, N, GA
PLIN2	NASH (D, T)	Lipid droplet degradation, cellular response to glucose starvation, lipid storage, long-chain fatty acid transport, positive regulation of sequestering of triglyceride.	CSF, CM, C, ER, Exo, ES, LD, MSF, N, PM
CPT-2	NAFLD/NASH (T)	Lipotoxicity, regulation of long fatty acid β-oxidation on IMM.	C, Exo, M, N
p62	NAFLD/NASH/NASH-HCC (D)	Autophagy, Ferroptosis, Microautophagy, Nrf2-mediated oxidative stress response, senescence pathway, brown fat cell proliferation.	AL, AV, N, C, ER IMBO, L, M
CNPY2	NASH HCC (D)	Cell death, outgrowth of neuritis, obesity, regulation of low-density lipoprotein particle clearance.	C, PM, N
CACHD1	NASH-HCC (D)	Potential calcium channel and chemotaxis receptor, interacts with Wnt-receptors, participates in protein folding, unfolded protein response, autophagy, apoptosis, cytoskeleton organization, neurogenesis.	C
LARP1	NASH-HCC (D)	Proliferation, positive regulation of macroautophagy, regulation of translation, TOR signaling cascade (negative regulator of autophagy).	C, N
Arginine	NASH-HCC (D)	Urea cycle, proliferation, cell viability, cell signaling, exocytosis, cytotoxicity, macrophage classical activation signaling pathway, phagosome maturation.	
Leucine	NASH HCC (D)	Phosphorylation, signaling, proliferation, premature senescence, cell death, inhibition of cell growth, angiogenesis.	
Indole-3- propionic acid	NASH-HCC (D)	Cell death, deamination metabolite of tryptophan maintaining intestinal homeostasis.	

Abbreviations: AG, Azurophil granules; AP, autophagosome; AV, Autophagic vacuoles; C, cytoplasm; CSF, Cell surface; CM, cellular membrane; DRLRF, detergent resistant lipid raft fraction ER, endoplasmic reticulum; ES, extracellular space, EM, extracellular matrix, Ex, exosomes; GA, Golgi apparatus; IMBO, intracellular membrane-bounded organelle; LD, lipid droplets; L, Lysosome; MOM, Mitochondrial outer membrane; MSF. Microsomal fraction; N, nucleus; P, peroxisomes; PM, plasma membrane; R, ribosome; SG, secretory granules.
